# Associations between Prenatal Exposure to Black Carbon and Memory Domains in Urban Children: Modification by Sex and Prenatal Stress

**DOI:** 10.1371/journal.pone.0142492

**Published:** 2015-11-06

**Authors:** Whitney J. Cowell, David C. Bellinger, Brent A. Coull, Chris Gennings, Robert O. Wright, Rosalind J. Wright

**Affiliations:** 1 Department of Environmental Health Sciences, Mailman School of Public Health, Columbia University, New York, New York, United States of America; 2 Children’s Hospital Boston, Boston, Massachusetts, United States of America; 3 Department of Environmental Health, Harvard School of Public Health, Boston, Massachusetts, United States of America; 4 Department of Biostatistics, Harvard School of Public Health, Boston, Massachusetts, United States of America; 5 Department of Preventive Medicine, Icahn School of Medicine at Mount Sinai, New York, New York, United States of America; 6 Kravis Children’s Hospital, Department of Pediatrics, Icahn School of Medicine at Mount Sinai, New York, New York, United States of America; 7 Mindich Child Health & Development Institute, Icahn School of Medicine at Mount Sinai, New York, New York, United States of America; University of Cincinnati, UNITED STATES

## Abstract

**Background:**

Whether fetal neurodevelopment is disrupted by traffic-related air pollution is uncertain. Animal studies suggest that chemical and non-chemical stressors interact to impact neurodevelopment, and that this association is further modified by sex.

**Objectives:**

To examine associations between prenatal traffic-related black carbon exposure, prenatal stress, and sex with children’s memory and learning.

**Methods:**

Analyses included N = 258 mother-child dyads enrolled in a Boston, Massachusetts pregnancy cohort. Black carbon exposure was estimated using a validated spatiotemporal land-use regression model. Prenatal stress was measured using the Crisis in Family Systems-Revised survey of negative life events. The Wide Range Assessment of Memory and Learning (WRAML2) was administered at age 6 years; outcomes included the General Memory Index and its component indices [Verbal, Visual, and Attention Concentration]. Relationships between black carbon and WRAML2 index scores were examined using multivariable-adjusted linear regression including effect modification by stress and sex.

**Results:**

Mothers were primarily minorities (60% Hispanic, 26% Black); 67% had ≤12 years of education. The main effect for black carbon was not significant for any WRAML2 index; however, in stratified analyses, among boys with high exposure to prenatal stress, Attention Concentration Index scores were on average 9.5 points lower for those with high compared to low prenatal black carbon exposure (*P*_*3-way interaction*_ = 0.04).

**Conclusion:**

The associations between prenatal exposure to black carbon and stress with children’s memory scores were stronger in boys than in girls. Studies assessing complex interactions may more fully characterize health risks and, in particular, identify vulnerable subgroups.

## Introduction

Black carbon (BC) is classified as near ultrafine and fine particulate matter composed of an elemental carbon core with adsorbed organic and inorganic chemical species [1]. In developed countries, traffic-related diesel exhaust is the principal source of airborne BC, resulting in nearly ubiquitous human exposure [1]. In addition to robust associations with multiple cardiopulmonary endpoints, recent research suggests the central nervous system is susceptible to the damaging effects of particulate air pollution, with animal and human studies demonstrating effects on neurodegenerative endpoints, including cognitive decline and neuropathology characteristic of memory and motor impairment [2]. Likewise, emerging results from epidemiological studies by our group and others have linked early life exposure to various constituents of air pollution, including BC, elemental carbon, polycyclic aromatic hydrocarbons, and particulate matter with disrupted cognitive, emotional, and behavioral development among children [3–8]. To date, the neurodevelopmental effects of BC exposure starting *in utero* remain largely unexplored.

Mounting epidemiologic and toxicologic evidence supports a framework in which susceptibility to the adverse neurodevelopmental effects of environmental chemicals is enhanced by concurrent or consecutive exposure to psychosocial stressors and stress correlates [9, 10]. Research suggests psychosocial stress and air pollution act on common biological systems (e.g., innate immune pathways, oxidative stress) in the developing brain [11]. Additionally, substantial evidence from the human and animal literature suggests that psychosocial stress and chemical toxicants elicit sex-specific effects on the developing brain, with boys typically demonstrating greater sensitivity to early life exposures relative to girls [3, 12–16]. These findings highlight the importance of examining the social environment and sex-specific differences when studying neurodevelopmental endpoints and support National Institute of Health policies that mandate clinical, animal and cellular-based research report how a study’s design will account for potential sex differences [17].

Moreover, neurodevelopmental studies in animals have identified complex interactions (toxicant×stress, toxicant ×sex, sex ×stress, and stress ×toxicant ×sex), even in the absence of significant independent effects [18, 19]. For example, a recent rodent study by Bolton et al. [20] observed joint effects of air pollution and prenatal stress on impaired cognitive function and increased expression of innate immune recognition genes only among male offspring. These findings suggest research assessing complex interactions may more accurately identify vulnerable subpopulations and more fully characterize health risks.

Despite the apparent intersection of these relationships, the interaction between prenatal exposure to BC, a surrogate of traffic particles, psychosocial stress and sex has not been previously examined in a human population. In the present study, we leveraged an existing ethnically diverse urban sample of pregnant women to assess the relationship between prenatal BC exposure and domain-specific memory and learning outcomes in early school-aged children. We additionally examined modification of this relationship by prenatal stress and infant sex. Our focus on memory and learning domains was informed by research demonstrating diesel exhaust preferentially targets brain regions essential for information encoding, storage and retrieval, including the hippocampus [21].

## Methods

### Study population

Data were collected as part of the Asthma Coalition on Community, Environment, and Social Stress project, a longitudinal pregnancy cohort [22]. Between August 2002 and December 2009 English and Spanish-speaking women (≥18 years old) receiving prenatal care at two Boston hospitals and their affiliated clinics were approached by trained bilingual research staff; among women approached on selected clinic days, 500 of those who were eligible (78.1%) were enrolled in mid to late pregnancy (28.4±7.9 weeks gestation) of which 455 gave birth to a live-born infant and continued follow-up. Previous analyses have demonstrated no significant differences for race/ethnicity, education or income between eligible women who enrolled and those who declined participation [23]. At age 6 years, 310 families were re-contacted to participate in neurodevelopmental testing, of which 300 completed testing.

### Ethics information

Written informed consent was obtained from mothers and assent was obtained for children ≥7 years of age. Study procedures were approved by the human studies committees at the Brigham and Women’s Hospital and Boston Medical Center.

### Prenatal black carbon: primary exposure

We used a previously validated spatiotemporal land-use regression model to estimate prenatal exposure to BC based on maternal residence over the entire pregnancy, taking into account moves. The model predicts 24-hour measures of BC based on over 6,000 measurements collected on more than 2,000 unique exposure days at 82 monitoring locations throughout the greater Boston area. The overall prenatal exposure level was calculated by averaging daily estimates throughout the duration of pregnancy. A detailed description of the land use regression model is provided elsewhere [3, 24]. Briefly, predictions were based on: BC level at a stationary central monitor, meteorological conditions, daily characteristics (e.g. weekday/weekend), and geographic information system-based measures, such as percent urbanization, distance to a major roadway, population and traffic densities. Traffic density was calculated as the product of cumulative traffic counts on road segments within 100 meters of the home and the length of each road segment. Splines were fit to permit non-linear relationships between predictors and BC and thin-plate splines were applied to capture additional spatial variability. Separate models were fit to represent the cold (November-April) and warm (May-October) months. The high predictive capacity of the model is demonstrated by a coefficient of determination (R^2^) over both seasons of 0.82.

### Childhood memory & learning outcomes

The Wide Range Assessment of Memory and Learning-Second Edition (WRAML2) [25] was administered to children at approximately 6 years of age. This test battery provides a dynamic and inclusive view of memory functioning. Raw scores are calculated for 6 core subtests, which are subsequently scaled by age to reflect developmental changes, summed based on the results of factor analytic studies, and standardized against a normative population to yield 3 core indices of memory function: the Attention Concentration Index, the Verbal Memory Index, and the Visual Memory Index. These 3 core indices sum to form the General Memory Index. Each index has a mean of 100 and standard deviation of 15 with lower scores indicating poorer function. The instrument’s external validity has been confirmed by comparison against several established neurodevelopmental batteries of memory and general cognitive ability, including the: Wechsler Memory Scales-III (WMS-III), Children’s Memory Scales (CMS), Test of Memory and Learning (TOMAL), and California Verbal Learning Test (CVLT). Moderate to high correlations were found on all comparisons, demonstrating moderate convergent and discriminant validity [25].

### Effect modifiers

#### Sex

Child sex was based on maternal report.

#### Prenatal stress

Prenatal stress was measured by maternal scores on the Crisis in Family Systems-Revised survey (CRYSIS-R) administered within 2 weeks of enrollment [26, 27]. This English- and Spanish-validated instrument provides information on life events experienced across 11 domains (e.g., financial, relationships, violence, housing issues, discrimination/prejudice, etc.) which mothers endorse as positive, negative, or neutral based on experiences over the prior 6 months. The number of domains for which one or more negative life event was endorsed were summed to create a continuous negative life event domain score with higher scores indicating greater stress. This approach is supported by evidence indicating vulnerability to the adverse psychological and physiological effects of stress increases with the number of distinct domains across which it is experienced [28].

### Other Covariates

We considered a number of standard control variables and potential confounders previously identified as being related to air pollution or stress exposure and cognitive development. Maternal age, race/ethnicity, education and smoking status were obtained by questionnaire during the prenatal period. Mothers were classified as prenatal smokers if they endorsed this behavior at either enrollment or during the 3^rd^ trimester follow-up. Data on birth weight were collected from labor and delivery record review; gestational age was calculated based on mother’s report of last menstrual period and updated using obstetrical estimates from medical record review at delivery if discrepant. We calculated z-scores to represent birth weight adjusted for gestational age using national United States reference values following methods developed to account for the non-linear relationship between these variables [29].

### Statistical analysis

If the pregnancy resulted in twin births the second born twin was excluded (n = 5) from the 300 completing neurodevelopmental testing. Dyads were additionally excluded if they were missing prenatal BC or prenatal stress (n = 34) or if the child had an incomplete WRAML2 test (n = 3), resulting in a final analytic sample of n = 258. Notably, those completing neurodevelopmental testing and included in these analyses did not differ from the overall sample on maternal age (27.4±5.7 vs. 27.0±6.1 years, respectively), race (26% black, 60% Hispanic vs. 28% black, 60% Hispanic, respectively) or education level (67% ≤12 years vs. 66% ≤12 years, respectively). We used multivariable linear regression to examine associations between prenatal BC exposure levels and child memory outcomes, adjusting for maternal race/ethnicity, educational status and smoking status during pregnancy, child age at exam, and birth weight for gestational age z-score. We considered each WRAML2 index in a separate model and examined effect modification by stress in stratified analyses and by fitting a 2-way interaction term (BC× prenatal stress). To aid interpretability, we created a categorical stress variable based on a tertile split (negative life event domain score: 0–1, 2–3, >3) and evaluated prenatal BC exposure per an interquartile range (IQR) increase (0.21 μg/m^3^). We examined effect modification by sex using stratified models and by fitting interaction terms. Specifically, we examined pairwise and three-way interactions: 1) BC× sex, 2) 2-way BC× prenatal stress within child sex strata, and 3) BC × stress ×sex. We included lower-order terms in all models and assessed statistical significance at a level of p<0.05. We considered mediation by birth weight for gestational age in secondary analyses as this factor may be on the causal pathway between prenatal exposure to air pollution and disrupted neurodevelopment [30–32]. We performed all analyses using SAS (version 9.4, Cary, North Carolina).

## Results

Table 1 presents summary statistics for the maternal-child dyads; distributions are provided for the sample as a whole and stratified by sex and prenatal stress level. The majority of mothers were ethnic minorities (60% Hispanic, 26% black), had ≤ 12 years of education (67%), and reported never smoking during pregnancy (85%). Children had a mean age of 6.6±1.1 years. Prenatal BC was approximately normally distributed with a median of 0.40 μg/m^3^ and range from 0.11 to 1.10 μg/m^3^. Prenatal stress was modestly correlated with BC exposure level (r_s_ = 0.17, p = 0.01). BC and covariate distributions did not significantly differ by sex or prenatal stress level, with the following exceptions: on average, girls (6.8±1.1 years) were older than boys (6.5±1.0 years) at the time of neurodevelopmental testing and girls performed better (98.8±14.4) than boys (94.8±14.8) on the Verbal Memory Index.

**Table 1:** ACCESS Participant Characteristics Stratified by Sex and Prenatal Stress.

Characteristic	Total (n = 258^a^)	Boys (n = 145)			Girls (n = 113)		
		Low Stress	Moderate Stress	High Stress	Low Stress	Moderate Stress	High Stress
**Child sex** [n (%)]							
Boys	145 (56)	61 (24)	48 (19)	36 (14)			
Girls	113 (44)				42 (16)	34 (13)	37 (14)
**Race/ethnicity** [n (%)]							
Black	68 (26)	20 (29)	12 (18)	6 (9)	4 (6)	10 (15)	16 (24)
Hispanic	155 (60)	31 (20)	30 (19)	22 (14)	32 (21)	22 (14)	18 (12)
White/other	35 (14)	10 (29)	6 (17)	8 (23)	6 (17)	2 (6)	3 (9)
**Maternal education** [n (%)]							
≤12 years	174 (67)	36 (21)	34 (20)	25 (14)	30 (17)	24 (14)	25 (14)
>12 years	84 (33)	25 (30)	14 (17)	11 (13)	12 (14)	10 (12)	12 (14)
**Smoking during pregnancy** [n (%)]							
No	220 (85)	50 (23)	45 (20)	31 (14)	39 (18)	29 (13)	26 (12)
Yes	38 (15)	11 (29)	3 (8)	5 (13)	3 (8)	5 (13)	11 (29)
**Maternal age** ^b^ (yrs) (mean±SD)	27.4±5.7	26.8±5.5	27.1±6.2	27.3±5.0	28.5±5.8	27.2±5.9	27.6±5.7
**BW percentile for GA** (mean±SD)	47.0±29.9	46.2±30.7	54.7±29.8	41.3±29.2	47.0±29.9	43.2±28.7	47.5±30.8
**Prenatal stress** ^c^ [median (IQR)]	2 (1–4)	1 (0–1)	2.5 (2–3)	5 (4–6)	1 (0–1)	2 (2–3)	5 (4–6)
**Prenatal BC** (μg/m^3^) [median (IQR)]	0.4 (0.3–0.5)	0.4 (0.3–0.5)	0.4 (0.3–0.5)	0.4 (0.3–0.5)	0.4 (0.3–0.5)	0.4 (0.3–0.6)	0.5 (0.4–0.6)
**Age at WRAML2** (yrs) (mean±SD)	6.6±1.0	6.4±0.9	6.5±1.1	6.5±0.9	6.6±1.0	6.9±1.2	6.8±1.0
**WRAML2 Indices** (mean±SD)							
General Memory	93.5±12.8	92.7±13.3	94.4±14.1	93.0±12.1	93.7±11.6	93.5±11.8	93.8±14.0
Attention Concentration	98.1±13.0	99.0±14.4	98.3±13.4	97.4±12.8	98.9±11.0	95.5±12.7	98.6±13.2
Verbal Memory	96.4±14.8	94.0±14.8	95.5±16.2	95.2±13.3	98.0±16.1	99.5±11.8	98.0±15.5
Visual Memory	90.7±14.9	91.0±14.9	93.4±17.1	92.2±14.7	88.5±13.9	90.8±14.3	87.6±13.3

Abbreviations: ACCESS, Asthma Coalition on Community, Environment, and Social Stress; BC, Black Carbon; BW, birth weight; GA, gestational age; WRAML2, Wide Range Assessment of Memory and Learning-2^nd^ edition.

^a^ Complete prenatal BC, prenatal stress, and WRAML2 data.

^b^Age at enrollment.

^c^ Negative life event domain scores from the Crisis in Family Systems-Revised survey; Range 0–8, stress categories defined using a tertile split (0–1, 2–3, >3).

Table 2 presents results from adjusted linear regression analyses including the main effect of BC, prenatal stress and infant sex, as well as the interactions between these variables for each WRAML2 index. In unadjusted and adjusted models, we did not observe statistically significant independent associations between BC exposure and each of the WRAML2 indices, albeit effects went in the anticipated direction based on prior literature, i.e., we observed lower scores on each index with increased BC exposure (Table 2). Two-way interactions between BC×prenatal stress were not significant, however, in sex-stratified models we observed a significant 2-way interaction for the Attention Concentration Index among boys (β_int_ = -10.52, 95% CI: -18.0, -3.00, n = 145) but not girls (β = 0.54, 95% CI: -6.88, 7.95, n = 113). Likewise, in sex- and stress-stratified analyses higher BC exposure was associated with a decrease in Attention Concentration Index score for boys born to mothers in the highest tertile of prenatal stress (β per IQR increase in BC = -6.03, 95% CI -12.8, 0.76, n = 36), but not for girls (β per IQR increase in BC = -1.86, 95% CI -8.4, 4.6, n = 37). In models examining the joint effect of BC, prenatal stress, and sex, we observed a significant 3-way interaction (*P*_*3-way interaction*_ = 0.04) between these variables for the Attention Concentration Index.

**Table 2:** Multivariable Linear Regression Models Examining Prenatal BC^a^ Exposure and Maternal Stress^b^ in Relation to WRAML2 Index Scores in Early School-Aged Urban Children, n = 258.

WRAML2 Index	Independent Variable	Main Effect Models^c^ ^,^ ^d^		BC x Stress x Male Model^d^ ^,^ ^e^	
		β±SE	p	β±SE	p
**General Memory**					
	BC	-0.55±1.0	0.58	-2.81±2.6	0.29
	Moderate Stress	1.13±1.9	0.55	-9.93±10.4	0.34
	High Stress	-0.06±2.0	0.98	1.10±10.2	0.91
	Male	-0.22±1.6	0.89	-9.38±7.8	0.23
	BC × Moderate Stress × Male			-2.10±5.0	0.67
	BC × High Stress × Male			-4.00±5.4	0.45
**Attention Concentration**					
	BC	-0.04±1.0	0.97	-2.37±2.6	0.36
	Moderate Stress	-1.75±1.9	0.36	-9.54±10.3	0.35
	High Stress	-1.36±2.0	0.49	-2.87±10.1	0.78
	Male	0.41±1.6	0.80	-12.61±7.7	0.10
	BC × Moderate Stress × Male			-1.73±4.9	0.72
	BC × High Stress × Male			-10.97±5.3	0.04
**Verbal Memory**					
	BC	-0.52±1.2	0.65	-0.54±3.0	0.86
	Moderate Stress	1.11±2.2	0.61	-6.14±11.9	0.61
	High Stress	0.31±2.3	0.89	8.52±11.7	0.47
	Male	-3.63±1.9	0.05	-5.73±8.9	0.52
	BC × Moderate Stress × Male			-1.95±5.7	0.73
	BC × High Stress × Male			2.75±6.2	0.66
**Visual Memory**					
	BC	-0.26±1.2	0.82	-3.41±3.1	0.26
	Moderate Stress	2.84±2.2	0.20	-7.61±12.0	0.53
	High Stress	0.24±2.3	0.92	-11.20±11.8	0.34
	Male	2.89±1.9	0.12	-4.30±9.1	0.64
	BC × Moderate Stress × Male			-2.53±5.8	0.66
	BC × High Stress × Male			-3.70±6.3	0.55

Abbreviations: BC, Black Carbon; WRAML2, Wide Range Assessment of Memory and Learning-2^nd^ edition.

^a^ Per an interquartile range increase (0.21 ug/m^3^) in BC.

^b^ Stress categories defined using a tertile split (0–1, 2–3, >3).

^c^ Main effect of BC, prenatal stress or sex in separate models.

^d^ Adjusted for: race/ethnicity, maternal high school education, maternal smoking during pregnancy, birth weight for gestational age z-score, and child age at exam.

^e^ 3-way interaction models included all lower order terms.

**Fig 1:**
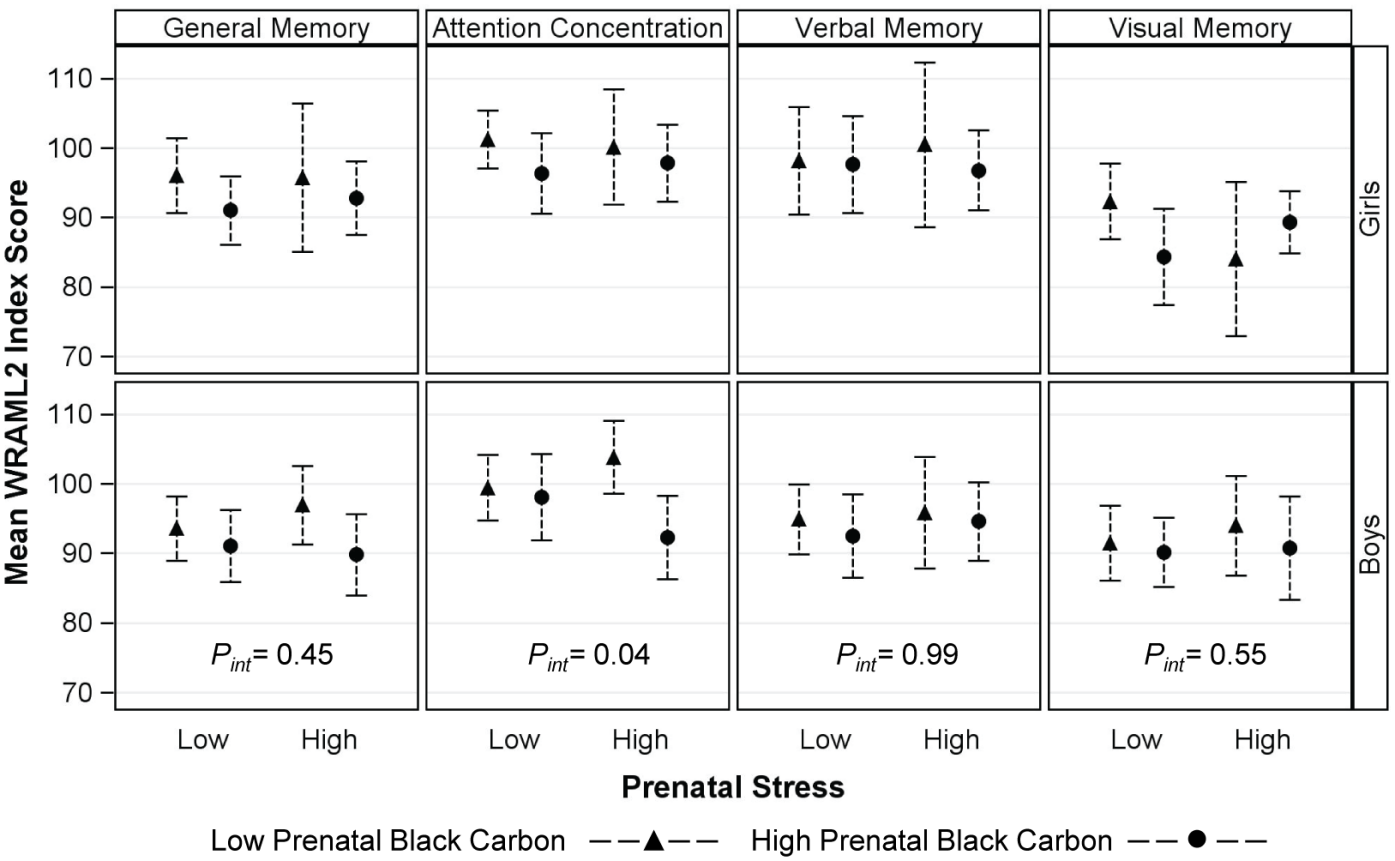
Mean WRAML2 Index Scores for Boys and Girls Stratified by Prenatal Stress and Black Carbon Exposure. Low and high prenatal stress are defined as negative life event domain scores ≤ 1 (lowest tertile) and >3 (highest tertile), respectively; BC is dichotomized using a median split (0.4 μg/m^3^). Error bars represent one standard deviation above and below the mean. Abbreviations: BC: black carbon; WRAML2: Wide Range Assessment of Memory and Learning-2nd edition. *P*_*int*_ represents the p-value from the 3-way interaction between child sex × prenatal stress × prenatal BC.

In order to better illustrate our findings, Fig 1 presents mean scores for each WRAML2 index stratified by BC (categorized into high/low based on the median of 0.4 μg/m^3^), prenatal stress (comparing the lowest to highest tertile) and sex. In adjusted models, we found that among boys with high exposure to prenatal stress, Attention Concentration Index scores were on average 9.5 points lower (95% CI -18.5, -0.6) for those with high compared to low prenatal BC exposure.

## Discussion

This is the first prospective epidemiological study to concurrently examine relationships between *in utero* BC exposure, prenatal stress and sex on neurodevelopment. This approach is consistent with recent National Institute of Health guidelines that mandate researchers address the potential modifying effect of sex in research going forward [17]. While we did not detect significant independent associations between BC or prenatal stress and memory or learning performance, effects on the Attention Concentration Index were in the expected direction (lower WRAML2 scores with increased BC or stress exposure).

Multiple animal and experimental studies demonstrate that air pollution effects on neurodevelopment begin *in utero* [33, 34], however, findings from human studies have been inconsistent [35–40]. This may be partially a result of variation in approaches for assessing exposure and outcomes. Fewer studies have examined the effects of prenatal stress on neurodevelopment. In a series of prospective experiments conducted in rhesus monkeys, Schneider et al. (2002) found offspring born to mothers exposed to random noise blasts during pregnancy, a construct designed to mimic daily episodic stress, presented with a unique behavioral profile characterized by shortened attention spans and reduced neuromotor capabilities during infancy. When examined during adolescence, the offspring prenatally exposed to stress during early gestation performed worse on cognitive tasks requiring working memory and attention shifting, compared to offspring exposed during mid-late gestation and controls. Interestingly, these researchers also found that a subset of offspring prenatally exposed to both noise blasts and alcohol, an established developmental neurotoxicant, showed the most impaired behavior, suggesting prenatal stress may provide a context within which the consequences of fetal alcohol exposure are greatest [41].

To our knowledge only one study has prospectively examined the relationship between prenatal stress and learning and memory in humans. Among a cohort of 112 children aged 6 years, Gutteling et al. (2006) found prenatal stress during pregnancy was negatively associated with performance on the Test of Memory and Learning Attention Concentration Index, while controlling for intelligence, sex and postnatal stress [42]. Notably, Gutteling et al. detected an association only with stress reported during early pregnancy (15–17 weeks) and did not examine sex-stratified models.

Psychosocial stress [43, 44] and particulate air pollution [12, 45, 46] are known to influence overlapping physiological pathways, including pro-inflammatory processes and oxidative stress, providing a plausible mechanism underlying our observed interactions [11]. Leading conceptual models suggest once absorbed into the systemic circulation, BC activates the maternal immune system, which subsequently triggers the placenta to produce cytokines and other inflammatory mediators with putative access to the fetal environment [2]. While the direct effects of increased immune activity on the fetal nervous system have not been elucidated, rodent models indicate peripherally circulating cytokines can activate microglia leading to neuroinflammation and oxidative stress in the brain [47]. Neuroinflammation has been shown to disturb programmed cell death, which is important for normal neuronal differentiation during mid-to-late pregnancy [48] and has been linked to reduced brain volume and thickness of the prefrontal cortex [49], which in turn are associated with attention deficit disorders [50]. Moreover, neuroimmune activation has been associated with disrupted myelination and synaptogenesis during pregnancy [48], which has adverse consequences for the acquisition and expression of spatial and trace memories [51, 52]. Within the hippocampus, a brain region with a central role in memory and learning, pro-inflammatory mediators have been shown to negatively influence neurogenesis, cell structure and organization, and production of brain-derived neurotrophic factor, a protein critical for memory-related synaptic plasticity [53, 54]. Human studies have shown children born to mothers experiencing infection-triggered immune activation during pregnancy are at increased risk for neuropsychiatric and neurodevelopmental disorders [55, 56]. Future epidemiological and animal studies specifically designed to examine the role of these and other factors are needed to fully understand the pathways underlying our observed associations.

The results of our 3-way interaction analysis suggest boys, but not girls, are susceptible to the joint effects of prenatal stress and traffic-related BC. These findings are consistent with epidemiologic evidence demonstrating boys are more susceptible to the adverse neurodevelopmental effects of stress [57] and an array of chemical toxicants, including BC [3], methylmercury [16, 58, 59], phthalates [60], organochlorines [61], bisphenol A [62], organophosphate pesticides [63], and metals [13]. Research using animal models further supports these findings. For example, male rodents exposed to stress *in utero* have been shown to have significantly increased hyperactivity [64] and impaired memory [65, 66] and learning [15] compared to controls. Similarly, a study examining prenatal exposure to concentrated ambient particles in mice found lateral ventricle dilation, a neuropathology characteristic of poor neurodevelopmental outcomes, was limited to male pups [12]. Moreover, results from a recent experimental study conducted by Bolton et al. (2013) found that among adult mice prenatally exposed to diesel exhaust particles and maternal stress (resource deprivation induced by nest material restriction), males exhibited greater memory deficits compared to unexposed controls [20]. Our findings are also consistent with research examining other developmental neurotoxicants, such as lead. For example, Cory-Slechta and colleagues [18, 67] have identified potentiated effects in rodent models, whereby the impact of lead on neurodevelopment varies by offspring sex and is unmasked only in the presence of stress. These findings underscore the importance of examining effect modification by sex and psychosocial stressors when assessing cognitive and behavioral endpoints as studies excluding these interactive effects may underestimate or even miss their detrimental developmental impact.

Our sex-specific findings may reflect developmental organization of the central nervous system and the brain circuitry underlying specific cognitive domains [68, 69]. For example, animal studies have identified specific regions (e.g., hypothalamus, cerebellum, hippocampus, amygdala) of male and female brains that differ in structure volumes, neuronal morphology, synaptic connections, and numbers of connecting fibers [70]. Post-mortem and imaging studies conducted among human populations further demonstrate significant differences in the relative volume and neural circuitry of structures central to learning and memory, including the hippocampus and prefrontal cortex [71, 72].

Several plausible explanations may underlie our findings of a significant interaction on the Attention Concentration Index, but not the other WRAML2 indices. The Attention Concentration Index provides a measure of executive attention, which is closely connected to the encoding and manipulation aspects of working memory. Notably, distinguishing between these processes is a major challenge for neuropsychologists and researchers [73]. While the WRAML2 includes an optional Working Memory Index, this subtest is moderately to highly (0.5–1.0) correlated with the Attention Concentration Index and factor analytic studies have identified a high degree of redundancy between the two [74]. At the neuroanatomical level, executive attention and working memory may be particularly vulnerable to neural insults as they depend not only on the integrity of discrete “memory centers”, but also the complex network of connecting pathways between them. Alternatively, our Attention Concentration findings may reflect an association between BC and attention processes not specifically measured by the WRAML2, such as alertness or vigilance, rather than impaired executive attention [75].

Major strengths of this study include its prospective design and enrollment of mother-child pairs from an urban, ethnically mixed community of lower socioeconomic status, a population that has traditionally been understudied despite disproportionate exposure to both environmental and social stressors. Additional strengths include our collection of extensive information on covariates, including data on a number of potential developmental neurotoxicants, and use of a validated land use regression model, which enabled examination of exposure over the duration of pregnancy. Unfortunately, we were not able to model exposure arising from locations other than the home where the mother may have spent time during pregnancy. However, we anticipate any exposure misclassification from this limitation would be non-differential resulting in a bias towards the null. Additional limitations include the potential for residual confounding by variables known to affect neurodevelopment that we were unable to examine (e.g., noise), and our use of BC, which may not be the only causal component of traffic-related air pollution. Furthermore, while we detected significant results, our study may have been underpowered to examine all 3-way interactions.

## Conclusion

Our results emphasize the importance of evaluating the effects of chemical stressors in the context of the social environment and highlight the need to explore effect modification by sex when studying neurocognitive and behavioral endpoints. We demonstrate boys exposed to both increased prenatal BC and prenatal stress may be a particularly vulnerable subpopulation. Studies assessing complex interactions may more accurately identify health risks and support the development of multidimensional strategies that encompass approaches for reducing the health impacts of both environmental and social stressors. Ultimately, we hope the emergence of cumulative approaches will aid in the elimination of health disparities by promoting an understanding of the mechanisms by which social conditions and environmental toxicants interrelate to give rise to vulnerable populations.

## Supporting Information

S1 Data File ACCESS Cohort Data. This excel workbook provides a minimal dataset including all variables analyzed in this analysis. A data dictionary is provided on worksheet two of the excel workbook.
(XLS)
